# Supplementary material for: Hidden information on protein function in censuses of proteome foldedness
Source: Nat Commun. 2022 Apr 14;13:1992. doi: 10.1038/s41467-022-29661-2 (PMC9010426; doi:10.1038/s41467-022-29661-2)
Supplement: Supplementary file 1 — Supplementary Information [file 41467_2022_29661_MOESM1_ESM.pdf]

Supplementary Information: Hidden information on protein function in censuses of proteome foldedness

D Hatters et al

This PDF file includes:

Supplementary Figure 1: TPE-MI reports on unfolding of recombinant β-lactoglobulin. Relates to Fig. 2.

Supplementary Figure 2: Residue and protein physicochemical properties. Relates to Fig. 3.

Supplementary Figure 3: Gene ontology terms enriched among multi-cluster proteins identified in published residue labelling dataset 4 [1]. Relates to Fig. 3.

Supplementary Figure 4: Gene ontology terms enriched among single cluster proteins. Relates to Fig. 3.

Supplementary Figure 5: Enrichment of chaperone machinery among multi-clustered proteins. Relates to Fig. 4.

Supplementary Figure 6: Detection of distinct HSPA8 and DNAJB1 conformations in the presence of urea. Relates to Fig. 4.

Supplementary Table 1: Published proteome stability dataset details

Supplementary Table 2: Summary of cysteine residues within multi-cluster chaperones which exhibited decreased reactivity during denaturation (cluster 4). Relates to Fig. 6.

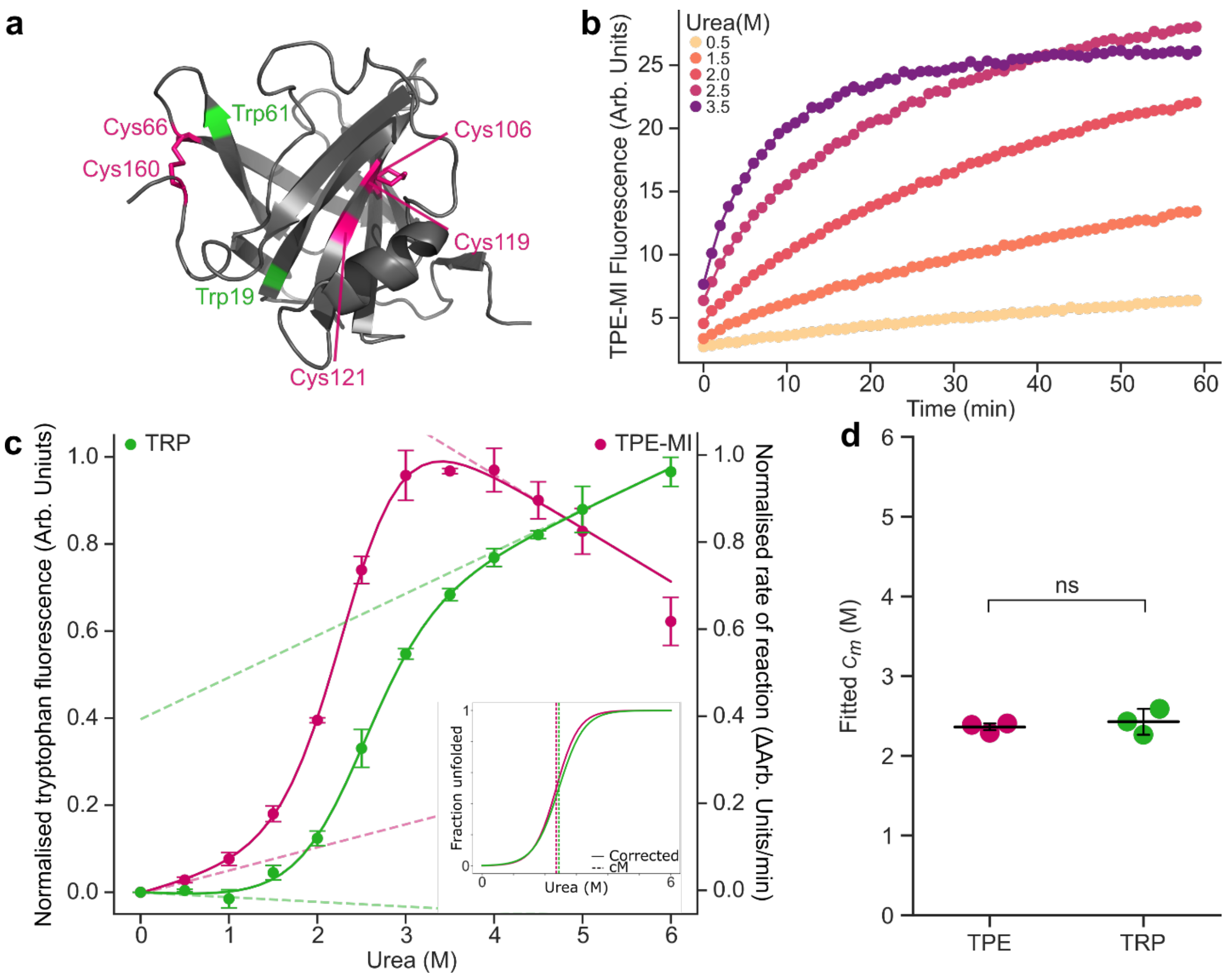

Supplementary Figure 1: TPE-MI reports on unfolding of recombinant β-lactoglobulin. Relates to Fig. 2. **a** Structure of β-lactoglobulin, a model globular protein, adapted from PDB entry 1CJ5[2]. Pertinent residues are highlighted in magenta (cysteine) and green (tryptophan). **b** Chemical denaturation of recombinant β-lactoglobulin in the presence of TPE-MI. Samples were equilibrated for 4 hours at room temperature before addition of TPE-MI, and TPE-MI fluorescence was monitored every 60 sec for 1 h. **c** The initial rate of reaction is calculated from B via linear regression and fitted to a denaturation curve. Curve is compared to intrinsic tryptophan fluorescence, also fitted to a two-state denaturation model. Dotted lines show linear pre- and post-transition components of the two-state model. Inset depicts the fitted two-state denaturation curve following pre- and post-transition correction. **d** Fitted C_m derived from C, compared using two-tailed t-test in GraphPad Prism. Exact statistical details are provided in Supplementary Data 1. In panels B – D, data shown are means ± S.D. of 3 replicates, and are representative of 2 independent experiments. Source data are provided as a Source Data file.

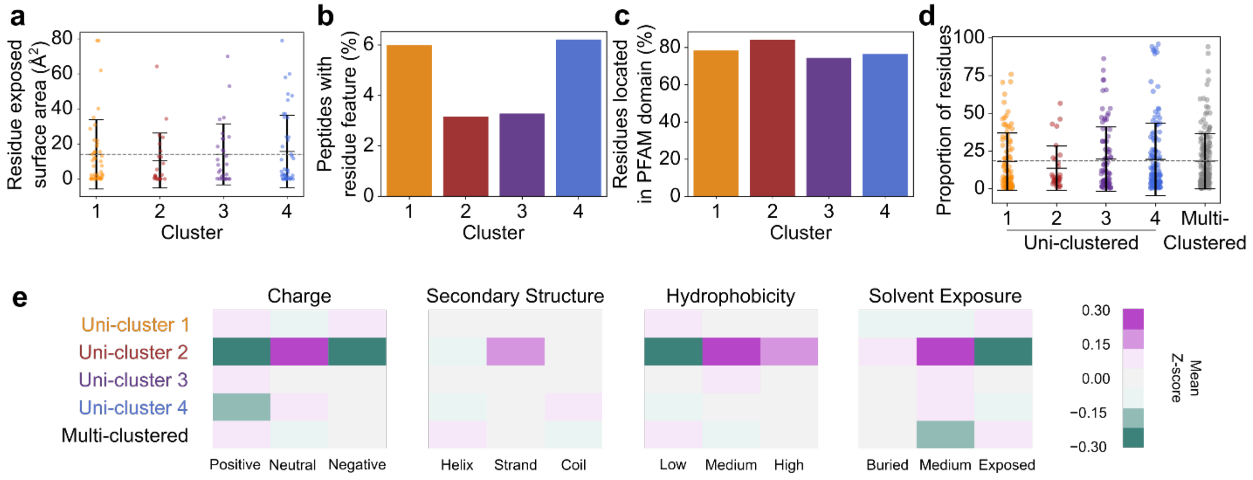

Supplementary Figure 2: Residue and protein physicochemical properties. Relates to Fig. 3. Individual cysteine residues contained within clustered peptides were assessed for (**a**) their relative surface exposure in experimentally determined structures available via the Protein Data Bank, (**b**) the proportion of residues annotated as a functional feature in UniProt, and (**c**) the proportion of residues located within curated PFAM domains. **d** The proportion of disordered residues in proteins associated with each cluster as predicted by IUPred2. **e** Mean *z*-score for predicted or extracted physiochemical features according to protein amino acid composition. Panels A and D show individual protein datapoints overlayed with mean ± S.D. Mean of clustered peptides (**a**) or combined uni-clustered proteins (**d**) is shown as dotted grey line. Exact statistical details including *n* and *p*-values are provided for panels **a** and **d** in Supplementary Data 1. Source data are provided as a Source Data file.

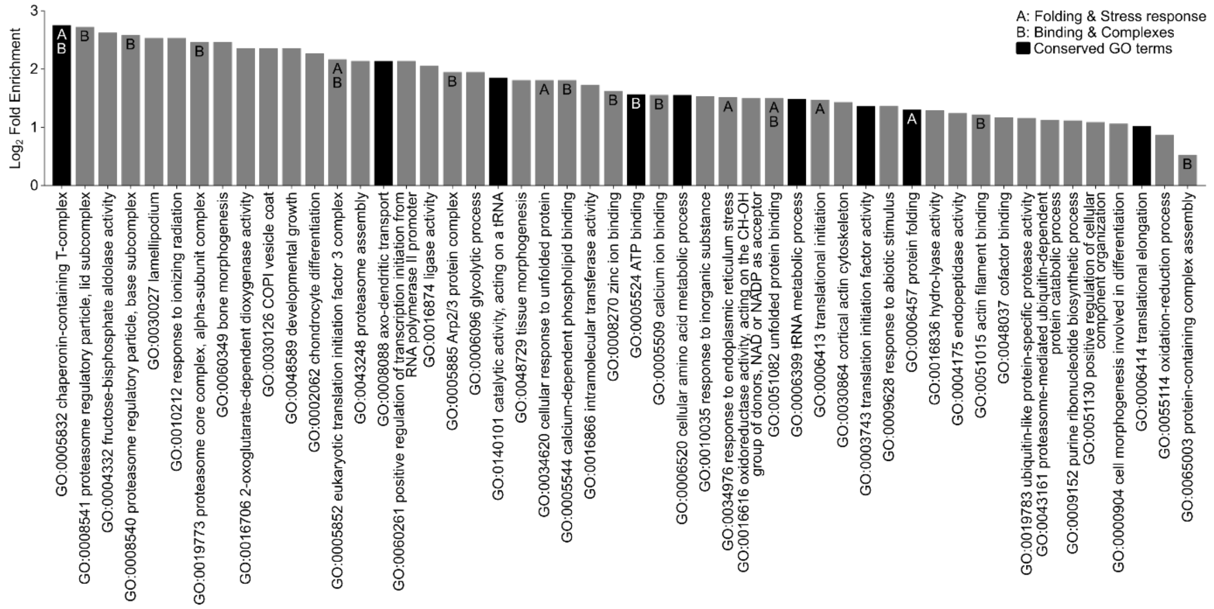

Supplementary Figure 3: Gene ontology terms enriched among multi-cluster proteins identified in published residue labelling dataset 4 [1]. Relates to Fig. 3. Enrichment determined using Panther GOSlim Fisher's overrepresentation test with false-discovery rate correction. Common themes are denoted: A = protein folding and stress response, B = binding and complexes. Dark bars denote exact terms found to also be enriched among multi-cluster proteins in the TPE-MI dataset.

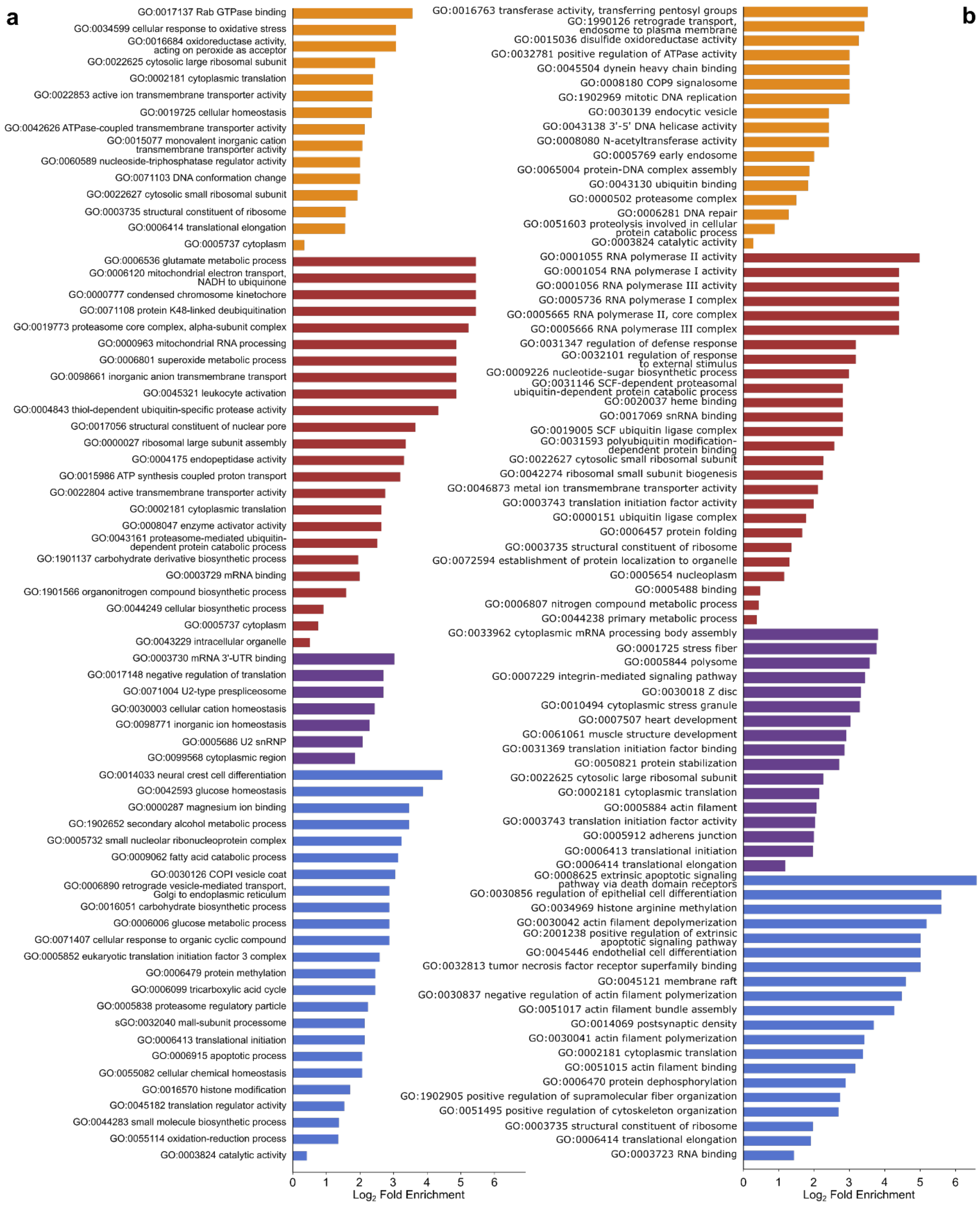

Supplementary Figure 4: Gene ontology terms enriched among uni-clustered proteins. Relates to Fig. 3. Enrichment for (***a***) TPE-MI dataset and (***b***) dataset 4 [1] clustered proteins determined using Panther GOSlim Fisher's overrepresentation test with false-discovery rate correction. Proteins associated with each cluster were compared against the background set of identified proteins. Shown are the outer-most terms for each hierarchical GO family which was significantly enriched ($p < 0.05$), with bars colored according to the cluster with which they were associated (orange, red, purple and blue correspond to clusters 1 – 4 respectively).

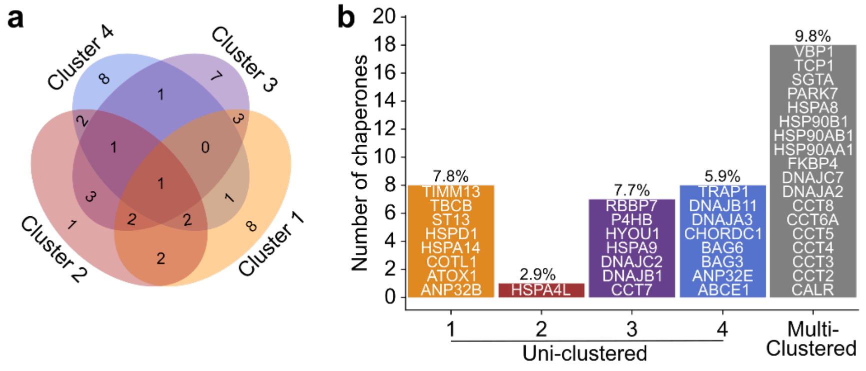

Supplementary Figure 5: Enrichment of chaperone machinery among multi-clustered proteins. Relates to Fig. 4. **a** Venn diagram depicting proportion of chaperone proteins for which peptides were found in each cluster combination. **b** Number and proportion of proteins in each cluster associated with “chaperone-mediated protein folding” gene ontology term (GO:0061077). Gene names for individual proteins are listed within the bars.

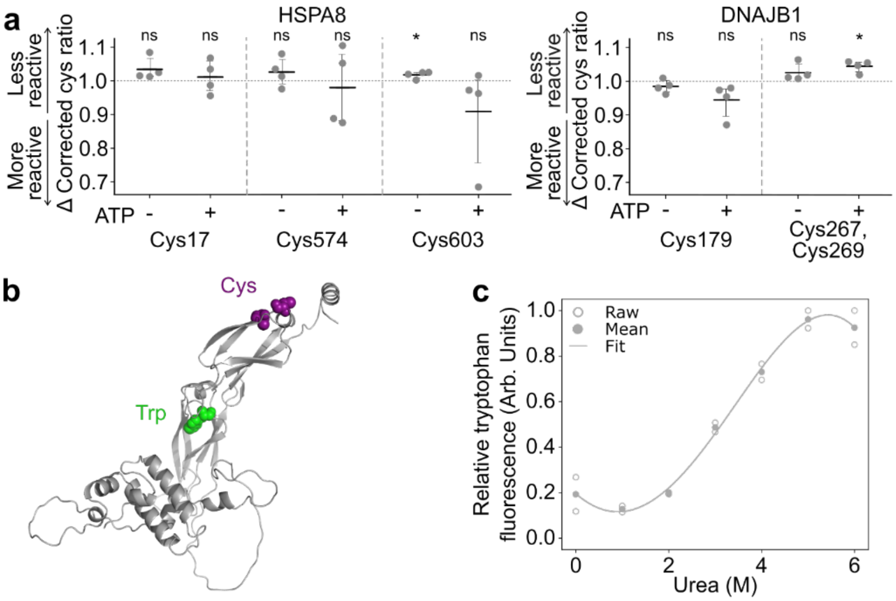

Supplementary Figure 6: Detection of distinct HSPA8 and DNAJB1 conformations in the presence of urea. a Change in cysteine reactivity of peptides derived from human HSPA8 and DNAJB1 when denatured together with MDH2 in 6 M urea, in the absence or presence of exogenous ATP prior to TPE-MI labelling. Means ± S.D. (*n*=4 biological replicates) are shown, and deviations from the expected mean of 1 were tested using a one-sample t-test (* denotes $p < 0.05$, ns denotes $p > 0.05$). Exact *p*-values are provided in Supplementary Data 1. **b** Ribbon structure of DNAJB1 (P25685) as predicted by Alphafold v1 [3]. Cysteine residues assigned to cluster 3 are colored purple, and tryptophan residues are colored in green. **c** Intrinsic tryptophan fluorescence was measured for DNAJB1 following denaturation with urea at concentrations from 0 – 6M. Normalized fluorescence values were then fitted to a two-state unfolding curve. Raw data (open) and mean (closed) values for two biological replicates are shown. Source data are provided as a Source Data file.

Supplementary Table 1: Published proteome stability dataset details.

Publication details	Reference	Technique	Species	Sample type	Dataset ID	Dataset filename(s)
Leuenberger et al 2017, Science 355: 7825	4	Proteolysis	Human	HeLa	1	aai7825_Leuenberger_Table-S3
Ogburn et al 2017, J Proteome Res. 16: 4073	5	Residue labelling	Human	MCF7	2, 3	pr7b00442_si_003<br>pr7b00442_si_004
Walker et al 2019, PNAS 26: 6081	1	Residue labelling	Human	HCA2-hTert	4	pnas.1819851116.sd04
Roberts et al 2016, J Proteome Res. 15: 4731	6	Residue labelling	Mouse	Brain tissue	5, 6	pr6b00927_si_003
Jarzab et al 2020, Nat Methods. 17: 495	7	Solubility	Human, Mouse	HepG2, Jurkat, K562, BMDC	7, 8, 9, 16, 17, 20	41592_2020_801_MOESM4_ESM
Becher et al 2016, Nat Chem Biol. 12: 908	8	Solubility	Human	HepG2	10	41589_2016_BFnchembio2185_MOESM256_ESM
Franken et al 2015, Nat Protoc. 10: 1567	9	Solubility	Human	K562	11	41596_2015_BFnprot2015101_MOESM411_ESM
Miettinen et al 2018, EMBO J. 37: e98359	10	Solubility	Human	MCF-7	12	embj201798359-sup-0003-tableev2
Savitski et al 2018, Cell. 173: 260	11	Solubility	Human	Jurkat, T-cells	13, 14	Fig5_SD6_reference_melting_curves
Ball et al 2020, Commun Biol. 3: 75	12	Solubility	Human	K562	15	42003_2020_795_MOESM2_ESM
Savitski et al 2014, Science. 346: 1255784	13	Solubility	Human	K562	18	Table_S4_Thermal_Profiling_Staurosporine_cell_extract, Table_S3_Thermal_Profiling_ATP_cell_extract
Sridharan et al 2019, Nat Commun. 10: 1155	14	Solubility	Human	Jurkat	19	41467_2019_9107_MOESM4_ESM

Supplementary Table 2: Summary of cysteine residues within multi-cluster chaperones which exhibited decreased reactivity during denaturation (cluster 4). Each row represents a unique cysteine-containing peptide. * denotes subunits of the T-complex protein Ring Complex (TRiC).

Protein	Cysteine(s)	Functional relevance	Reference
HSPA8	574	Located in the helix:helix interface of the helical lid domain (SBDα) required for chaperone function, implicated in non-specific client interaction.	15 16
	603	Located in the helix:helix interface of the helical lid domain (SBDα) required for chaperone function, implicated in non-specific client interaction.	
HSP90AA1	530	Located in 'middle domain', positioned within a group of residues (E528, Y529, Q532) shown to be important for client binding and chaperone function.	17
HSP90B1	576	Located in C-terminal domain responsible for dimerization, in a region responsible for communicating nucleotide status to the N-terminal domain.	18
CALR	105	Located within the globular N-terminal domain, shown to mediate peptide binding and chaperone function.	19,20
CCT2*	395	Located in the Intermediate–Equatorial domain hinge region, associated with intra-ring contact with neighboring CCT5 subunit in Z conformation when TRiC is in open state. CCT2 is also key subunit for substrate binding.	21,22
CCT3*	366	Located at the base of the apical domain, adjacent to substrate-binding surface.	
CCT6*	233	Located in the center of the apical domain, spanning opposing face to substrate-binding surface at the interface with TCP1, associated with intra-ring contact with neighboring CCT8 subunit when TRiC is in open state.	
CCT8*	244	Located at top of the apical domain, at the interface with CCT6 substrate-binding surface and associated with intra-ring contact with neighboring CCT6 subunit when TRiC is in open state.	
SGTA	149	Located within a tetratricopeptide repeat domain (TPR2), which together with TPR1 domain mediates interaction with HSC70, HSP0 and HSP90β.	23,24
PARK7	46	Located in the homodimer interface, necessary for chaperone action, within the region required for client binding.	25–27
	53	Required for dimerization and chaperone action, involved in interaction with HSPA5 (BiP), within the region required for client binding.	

SUPPLEMENTARY REFERENCES

1. Walker, E. J., Bettinger, J. Q., Welle, K. A., Hryhorenko, J. R. & Ghaemmaghami, S. Global analysis of methionine oxidation provides a census of folding stabilities for the human proteome. *PNAS* **116**, 6081–6090 (2019).
2. Kuwata, K. *et al.* Solution structure and dynamics of bovine β-lactoglobulin A. *Protein Science* **8**, 2541–2545 (1999).
3. Jumper, J. *et al.* Highly accurate protein structure prediction with AlphaFold. *Nature* **596**, 583–589 (2021).
4. Leuenberger, P. *et al.* Cell-wide analysis of protein thermal unfolding reveals determinants of thermostability. *Science* **355**, (2017).
5. Ogburn, R. N., Jin, L., Meng, H. & Fitzgerald, M. C. Discovery of Tamoxifen and N-Desmethyl Tamoxifen Protein Targets in MCF-7 Cells Using Large-Scale Protein Folding and Stability Measurements. *J. Proteome Res.* **16**, 4073–4085 (2017).
6. Roberts, J. H., Liu, F., Karnuta, J. M. & Fitzgerald, M. C. Discovery of age-related protein folding stability differences in the mouse brain proteome graphical abstract HHS public access. *J Proteome Res* **15**, 4731–4741 (2016).
7. Jarzab, A. *et al.* Meltome atlas—thermal proteome stability across the tree of life. *Nature Methods* **17**, 495–503 (2020).
8. Becher, I. *et al.* Thermal profiling reveals phenylalanine hydroxylase as an off-target of panobinostat. *Nature Chemical Biology* **12**, 908–910 (2016).
9. Franken, H. *et al.* Thermal proteome profiling for unbiased identification of direct and indirect drug targets using multiplexed quantitative mass spectrometry. *Nature Protocols* **10**, 1567–1593 (2015).
10. Miettinen, T. P. *et al.* Thermal proteome profiling of breast cancer cells reveals proteasomal activation by CDK4/6 inhibitor palbociclib. *The EMBO journal* e98359 (2018) doi:10.15252/embj.201798359.
11. Savitski, M. M. *et al.* Multiplexed proteome dynamics profiling reveals mechanisms controlling protein homeostasis. *Cell* **173**, 260-274.e25 (2018).
12. Ball, K. A. *et al.* An isothermal shift assay for proteome scale drug-target identification. *Communications Biology* **3**, 1–10 (2020).
13. Savitski, M. M. *et al.* Tracking cancer drugs in living cells by thermal profiling of the proteome. *Science* **346**, 1255784 (2014).
14. Sridharan, S. *et al.* Proteome-wide solubility and thermal stability profiling reveals distinct regulatory roles for ATP. *Nature Communications* **10**, 1155 (2019).
15. Jones, G. W. & Perrett, S. The C-terminal GGAP motif of Hsp70 mediates substrate recognition and stress response in yeast. *J Biol Chem* **293**, 17663–17675 (2018).
16. Schlecht, R., Erbse, A. H., Bukau, B. & Mayer, M. P. Mechanics of Hsp70 chaperones enables differential interaction with client proteins. *Nat Struct Mol Biol* **18**, 345–351 (2011).
17. Genest, O. *et al.* Uncovering a region of Hsp90 important for client binding in E. coli and chaperone function in yeast. *Mol Cell* **49**, 464–473 (2013).
18. Krukenberg, K. A., Street, T. O., Lavery, L. A. & Agard, D. A. Conformational dynamics of the molecular chaperone Hsp90. *Q Rev Biophys* **44**, 229–255 (2011).
19. Chouquet, A. *et al.* X-Ray Structure of the Human Calreticulin Globular Domain Reveals a Peptide-Binding Area and Suggests a Multi-Molecular Mechanism. *PLoS One* **6**, e17886 (2011).
20. Martin, V. *et al.* Identification by Mutational Analysis of Amino Acid Residues Essential in the Chaperone Function of Calreticulin *. *Journal of Biological Chemistry* **281**, 2338–2346 (2006).
21. Zang, Y. *et al.* Staggered ATP binding mechanism of eukaryotic chaperonin TRiC (CCT) revealed through high-resolution cryo-EM. *Nat Struct Mol Biol* **23**, 1083–1091 (2016).
22. Joachimiak, L. A., Walzthoeni, T., Liu, C. W., Aebersold, R. & Frydman, J. The Structural Basis of Substrate Recognition by the Eukaryotic Chaperonin TRiC/CCT. *Cell* **159**, 1042–1055 (2014).
23. Liu, F. H., Wu, S. J., Hu, S. M., Hsiao, C. D. & Wang, C. Specific interaction of the 70-kDa heat shock cognate protein with the tetratricopeptide repeats. *J Biol Chem* **274**, 34425–34432 (1999).
24. Liou, S.-T. & Wang, C. Small glutamine-rich tetratricopeptide repeat-containing protein is composed of three structural units with distinct functions. *Arch Biochem Biophys* **435**, 253–263 (2005).
25. Shendelman, S., Jonason, A., Martinat, C., Leete, T. & Abeliovich, A. DJ-1 Is a Redox-Dependent Molecular Chaperone That Inhibits α-Synuclein Aggregate Formation. *PLoS Biol* **2**, e362 (2004).
26. Lee, D. H. *et al.* PARK7 modulates autophagic proteolysis through binding to the N-terminally arginylated form of the molecular chaperone HSPA5. *Autophagy* **14**, 1870–1885 (2018).

27. McNally, R. S. *et al.* DJ-1 Enhances Cell Survival through the Binding of Cezanne, a Negative Regulator of NF-κB *. *Journal of Biological Chemistry* **286**, 4098–4106 (2011).
