# Supplementary material for: Hidden information on protein function in censuses of proteome foldedness
Source: Nat Commun. 2022 Apr 14;13:1992. doi: 10.1038/s41467-022-29661-2 (PMC9010426; doi:10.1038/s41467-022-29661-2)
Supplement: Supplementary file 3 — Description of Additional Supplementary Files [file 41467_2022_29661_MOESM3_ESM.pdf]

Description of Additional Supplementary Files

File name: Supplementary Data 1

Description: Statistical analyses summary

File name: Supplementary Data 2

Description: Supplementary dataset – lysate denaturation preprocessed peptide data

File name: Supplementary Data 3

Description: Supplementary dataset – recombinant client-binding assay preprocessed peptide data

File name: Supplementary Data 4

Description: Supplementary dataset – HSP70 inhibitor preprocessed peptide data
