# Supplementary material for: Hidden information on protein function in censuses of proteome foldedness
Source: Nat Commun. 2022 Apr 14;13:1992. doi: 10.1038/s41467-022-29661-2 (PMC9010426; doi:10.1038/s41467-022-29661-2)
Supplement: Supplementary file 8 — Reporting Summary [file 41467_2022_29661_MOESM8_ESM.pdf]

nature research

Corresponding author(s): Danny Hatters

Last updated by author(s): Mar 1, 2022

Reporting Summary

Nature Research wishes to improve the reproducibility of the work that we publish. This form provides structure for consistency and transparency in reporting. For further information on Nature Research policies, see our Editorial Policies and the Editorial Policy Checklist.

Statistics

For all statistical analyses, confirm that the following items are present in the figure legend, table legend, main text, or Methods section.

n/a	Confirmed	
☐	☒	The exact sample size (*n*) for each experimental group/condition, given as a discrete number and unit of measurement
☒	☐	A statement on whether measurements were taken from distinct samples or whether the same sample was measured repeatedly
☐	☒	The statistical test(s) used AND whether they are one- or two-sided<br>*Only common tests should be described solely by name; describe more complex techniques in the Methods section.*
☒	☐	A description of all covariates tested
☐	☒	A description of any assumptions or corrections, such as tests of normality and adjustment for multiple comparisons
☐	☒	A full description of the statistical parameters including central tendency (e.g. means) or other basic estimates (e.g. regression coefficient) AND variation (e.g. standard deviation) or associated estimates of uncertainty (e.g. confidence intervals)
☐	☒	For null hypothesis testing, the test statistic (e.g. *F*, *t*, *r*) with confidence intervals, effect sizes, degrees of freedom and *P* value noted<br>*Give P values as exact values whenever suitable.*
☒	☐	For Bayesian analysis, information on the choice of priors and Markov chain Monte Carlo settings
☒	☐	For hierarchical and complex designs, identification of the appropriate level for tests and full reporting of outcomes
☒	☐	Estimates of effect sizes (e.g. Cohen's *d*, Pearson's *r*), indicating how they were calculated

Our web collection on statistics for biologists contains articles on many of the points above.

Software and code

Policy information about availability of computer code

Data collection	Raw mass spectra were obtained using the Xcalibur instrument control software v 4.3.73.11 and Tune application v 3.3.2782.34<br>Protein feature data were programatically compiled on demand using custom analysis scripts available via 10.5281/zenodo.4287766 [https://doi.org/10.5281/zenodo.4287766] from the following database APIs:<br>- UniProt [https://www.uniprot.org/]<br>- PFAM [https://pfam.xfam.org/]<br>- Protein Data Bank [https://www.ebi.ac.uk/pdbe/]<br>- DSSP [https://swift.cmbi.umcn.nl/gv/dssp/]<br>- IUPred2A [https://iupred2a.elte.hu/]<br>- STRINGdb [https://string-db.org/api/]<br>- PantherGOSlim [http://pantherdb.org]<br>- iFeature [https://ifeature.erc.monash.edu/]
Data analysis	Proteome Discoverer v 2.1<br>MaxQuant v 1.6.3.4<br>GraphPad Prism v 8.4.3<br>Python v 3.8.2<br>SciPy v 1.5.0<br>Scikit-learn v0.23.1<br>Custom analysis scripts available from 10.5281/zenodo.4287766 [https://doi.org/10.5281/zenodo.4287766].

For manuscripts utilizing custom algorithms or software that are central to the research but not yet described in published literature, software must be made available to editors and reviewers. We strongly encourage code deposition in a community repository (e.g. GitHub). See the Nature Research guidelines for submitting code & software for further information.

Data

Policy information about availability of data

All manuscripts must include a data availability statement. This statement should provide the following information, where applicable:

- Accession codes, unique identifiers, or web links for publicly available datasets
- A list of figures that have associated raw data
- A description of any restrictions on data availability

The mass spectrometry proteomics data generated in this study have been deposited in the ProteomeXchange Consortium via the PRIDE partner repository database under accession codes PXD022587, PXD022640 and PXD030567 [http://proteomecentral.proteomexchange.org/].
All other data produced in this study are available from Zenodo via the DOI 10.5281/zenodo.4280620 [https://doi.org/10.5281/zenodo.4280620], and select summary datasets are also provided in the Supplementary Information and Source Data files.

Field-specific reporting

Please select the one below that is the best fit for your research. If you are not sure, read the appropriate sections before making your selection.

[x] Life sciences [ ] Behavioural & social sciences [ ] Ecological, evolutionary & environmental sciences

For a reference copy of the document with all sections, see nature.com/documents/nr-reporting-summary-flat.pdf

Life sciences study design

All studies must disclose on these points even when the disclosure is negative.

Sample size	Replicates were chosen based on what could be practically handled. We chose no fewer than three replicates for the key experiments, which enabled us to do basic statistical comparisons.
Data exclusions	Published proteome stability datasets garnered from the systematic literature search were excluded if they did not meet the following criteria: (i) reported primary experimental data for proteome-wide protein stability under control conditions, (ii) were derived from either human or mouse samples, and (iii) provided a publicly-accessible summary of the dataset. A total of 12 papers were found to meet these criteria. One dataset from the urea denaturation of lysate was removed during quality control due to a poor peptide quantification rate, whereby the mean number of quantified peptides was assessed via Grubbs test for outliers (two-sided test $p < 0.05$, corresponding to less than 15% of identified peptides quantified).
Replication	At least three biological replicates were performed for proteomics experiments, and two for recombinant protein experiments (which also included technical replicates).
Randomization	Homogenous cell populations were randomly allocated to individual treatment conditions to prevent batch growth effects. Sample labelling strategies for proteomics were not randomised, however did contain internal controls to account for batch effects.
Blinding	Proteomics spectra were acquired in a blinded fashion. Initial sample preparations and final analyses were not blinded, and were without any subjective measurements.

Reporting for specific materials, systems and methods

We require information from authors about some types of materials, experimental systems and methods used in many studies. Here, indicate whether each material, system or method listed is relevant to your study. If you are not sure if a list item applies to your research, read the appropriate section before selecting a response.

Materials & experimental systems

n/a	Involved in the study	
[x]	[ ]	Antibodies
[ ]	[x]	Eukaryotic cell lines
[x]	[ ]	Palaeontology and archaeology
[x]	[ ]	Animals and other organisms
[x]	[ ]	Human research participants
[x]	[ ]	Clinical data
[x]	[ ]	Dual use research of concern

Methods

n/a	Involved in the study	
[x]	[ ]	ChIP-seq
[x]	[ ]	Flow cytometry
[x]	[ ]	MRI-based neuroimaging

Eukaryotic cell lines

Policy information about cell lines

Cell line source(s)	Neuro2a from American Type Culture Collection
Authentication	None of the cells were authenticated
Mycoplasma contamination	Cells were tested negative for mycoplasma
Commonly misidentified lines (See ICLAC register)	None
